# Disección Aórtica Stanford A Crónica Asintomática Post Cirugía de Revascularización Coronaria

**DOI:** 10.47487/apcyccv.v1i1.15

**Published:** 2020-03-30

**Authors:** Juan Muñoz, Rosario Guzmán, Violeta Illatopa, Carlos Pereda

**Affiliations:** 1 Médico residente de Cardiología - Instituto Nacional Cardiovascular - INCOR EsSalud. Lima, Perú. Instituto Nacional Cardiovascular - INCOR EsSalud Lima Perú; 2 Servicio de Cardiología Clínica - Instituto Nacional Cardiovascular - INCOR EsSalud. Lima, Perú. Servicio de Cardiología Clínica Instituto Nacional Cardiovascular - INCOR EsSalud Lima Perú; 3 Servicio de Cardiología No Invasiva - Instituto Nacional Cardiovascular - INCOR EsSalud. Lima, Perú. Servicio de Cardiología No Invasiva nstituto Nacional Cardiovascular - INCOR EsSalud Lima Perú

**Keywords:** disección aórtica, cirugía de revascularización coronaria, aortic dissection, myocardial revascularization surgery

## Abstract

La disección aórtica Stanford A aguda rara vez hace una transición al estado crónico de forma natural, por su elevada mortalidad. Presentamos un caso raro de disección aórtica Stanford A crónica post cirugía, en un paciente que permaneció estable por más de 1 año, y posteriormente presentó dolor torácico.

## Abstract (en)

Acute Stanford A aortic dissection rarely makes a transition to the chronic state naturally, due to its high mortality. We present a rare case of chronic Stanford A aortic dissection post surgery, in a patient who remained stable for more than 1 year, and subsequently presented chest pain.

La disección aórtica es la rotura de la capa media de la aorta causada por una hemorragia intramural, que resulta en la separación de las capas de la pared, siendo la disrupción intimal generalmente la lesión de inicio.^1^ Se clasifica por pronóstico y consecuencias terapéuticas en Stanford tipos A y B, y según el tiempo en aguda (menos de 14 días), subaguda (15 a 90 días) y crónica (más de 90 días).^2^ La incidencia es de 3 a 6 casos por cada 100,000 personas, por año.^2^

## Descripción del Caso

Varón de 67 años, procedente de Piura, con antecedente de hipertensión arterial, dislipidemia, y 2 accidentes cerebrovasculares hace 1 y 6 años que no dejaron secuela. 1 año antes del inicio de síntomas presentó un infarto de miocardio tratado con cirugía de revascularización coronaria (arteria mamaria interna a descendente anterior y vena safena interna a marginal) en otro hospital. Asimismo, se detectó estenosis carotídea severa derecha que no fue tratada. 

**Figura 1:**
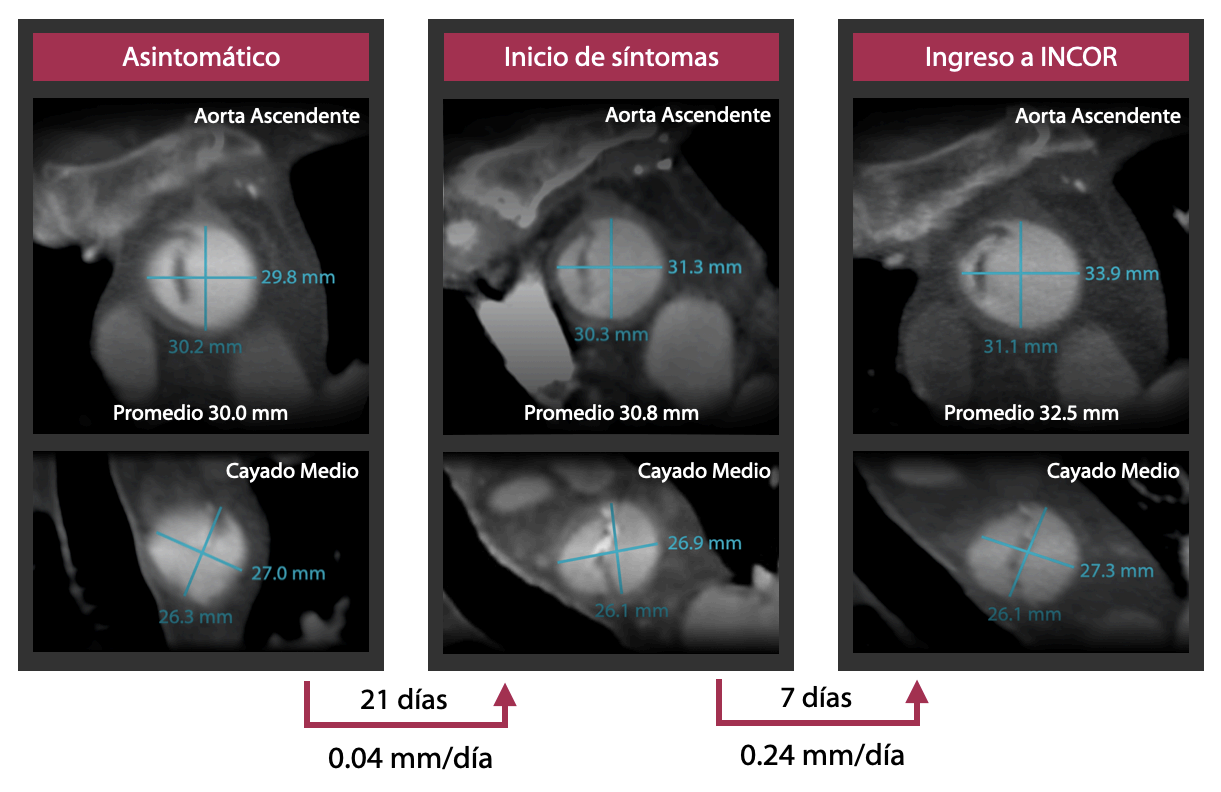
AngioTEM de aorta ascendente y cayado en estado asintomático, al inicio de síntomas y al ingreso a INCOR. Nótese la variación de los diámetros promedios en el tiempo.

El paciente acudió a un centro de diagnóstico a realizarse una angiotomografía espiral multicorte (angioTEM) de carótidas de control. En el estudio se observó incidentalmente un trazo de disección de aorta ascendente y cayado aórtico. (Figura 1) Sin embargo, por no presentar síntomas, fue enviado a casa.

Luego de 21 días, presentó dolor torácico súbito, lancinante, desgarrante, irradiado a espalda, y acompañado de síntomas neurovegetativos. Acudió a un hospital en Piura, en donde ingresa con presión arterial 149/83 mmHg, y frecuencia cardíaca (FC) de 61 lpm. Se le realizó un angioTEM de aorta (Figura 1) que evidenció la disección. Con exámenes complementarios se descartó infarto de miocardio. 

Siete días después del inicio del dolor, llegó referido al Instituto Nacional Cardiovascular - INCOR EsSalud, con dolor torácico persistente 4/10. Al examen físico se encontró presión arterial en brazo derecho de 110/70 y en brazo izquierdo de 100/60, ruidos cardíacos disminuidos en intensidad, y pulsos periféricos disminuidos en intensidad (+/+++) principalmente en miembros inferiores y brazo izquierdo. Dentro de los exámenes complementarios destacamos lo siguiente: El electrocardiograma mostró hemibloqueo anterior izquierdo sin otra alteración significativa. Los exámenes de laboratorio se encontraron dentro de límites normales. El ecocardiograma transtorácico reveló una fracción de eyección de 52%, y un flap de disección a nivel del tercio medio de la aorta ascendente que se continuaba a nivel del cayado. Finalmente, un nuevo angioTEM de aorta mostró una disección aórtica Stanford A que iniciaba a 65 mm del anillo aórtico y se extendía hasta el cayado. (Figura 1)

**Figura 2:**
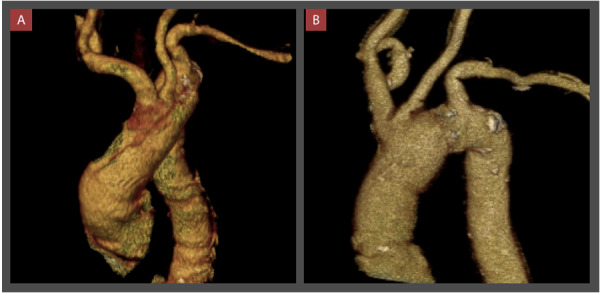
AngioTEM de aorta con reconstrucción 3D antes (A) y después (B) de la cirugía.

Se realizó una junta médica en la que se definió la necesidad de tratamiento quirúrgico. El procedimiento realizado fue el reemplazo de aorta ascendente y arco aórtico con dacrón n.° 28, con reimplante de vasos supraaórticos y endarterectomía de carótida derecha con parche de vena. (Figuras 2 y 3)

**Figura 3:**
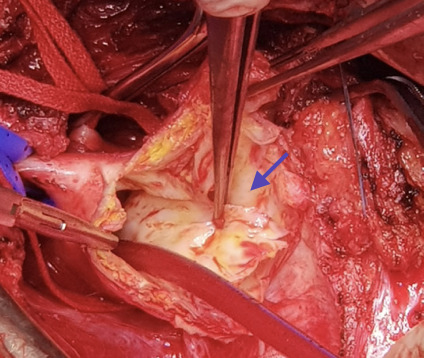
Cirugía de disección aórtica. Se evidencia el flap de la íntima (flecha azul).

En el postoperatorio, cursó con una infección respiratoria que respondió adecuadamente a antibioticoterapia por 7 días, y síndrome confusional agudo que también se resolvió. Evolucionó favorablemente y al alta se encontraba asintomático. En controles ambulatorios se ha mantenido estable.

## Discusión

La disección aórtica Stanford A aguda rara vez (<10%) hace una transición al estado crónico de forma natural, pues aproximadamente el 90% de los casos se tratan quirúrgicamente en su presentación inicial.^2^

**Figura 4:**
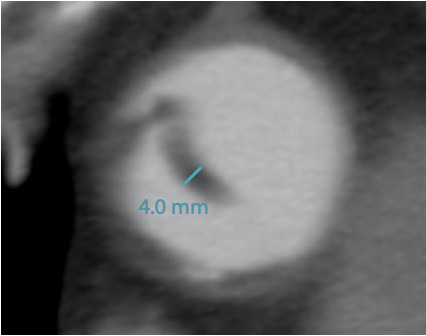
AngioTEM de aorta en vista axial que muestra el engrosamiento del flap (4 mm).

Ya que no existen estudios sobre la transición natural de la disección aórtica Stanford A hacia la fase crónica (por la alta mortalidad y tasa de complicaciones en la etapa aguda), extrapolamos los hallazgos de Peterss et al, quienes desde 1999 hasta 2015 hicieron seguimiento a 104 pacientes con disección aórtica Stanford B (de un total de 414 pacientes con disección aórtica) en el Instituto Aórtico del Hospital Yale New Haven.^2^ Según estos autores, los cambios en el engrosamiento del flap presentan una dinámica de decaimiento exponencial a lo largo del tiempo, con tasas marcadamente más altas en el estado agudo y subagudo. La estabilización en la tasa de engrosamiento se alcanza después de 83 días, y la meseta después de 235 días (aproximadamente 8 meses). En esta serie el grosor promedio del flap 1 año después del inicio de síntomas fue de 2.4 mm y en ningún caso el grosor fue mayor de 3.5 mm.^2^ Nuestro paciente presentó un flap con grosor de 4 mm (Figura 4), lo que sugiere que se trataba de una disección crónica.

El diámetro de la aorta también sufre cambios en el tiempo, con un rápido crecimiento en la etapa aguda (0.1-0.7 mm/día), un descenso en la subaguda (menor a 0.1 mm/día) que se estabiliza luego de 25 días, y una meseta en la etapa crónica que se alcanza después de 88 días.^2^ Si comparamos las tomografías de los días 19/07 (asintomático) y 09/08 (inicio de síntomas 21 días después), observamos que la variación del diámetro promedio de la aorta ascendente es de 0,04 mm/día, un cambio mínimo que es compatible con la fase crónica de la disección. Por otro lado, si comparamos las tomografías del 09/08 (inicio de síntomas) y 16/08 (ingreso a INCOR 7 días después), notaremos una variación de 0.24 mm/día, lo que sugiere reagudización del cuadro. (Figura 1)

De Jong et al. estudiaron los hallazgos de tomografía computarizada de las calcificaciones de pared aórtica en 69 pacientes con disección aórtica, encontrando que las calcificaciones de la capa media son más frecuentes en casos crónicos (52% vs 22%).^3^ Esta característica también estuvo presente en nuestro paciente como podemos observar en la Figura 1 donde se muestra calcio en el flap. Otro hallazgo usual en una disección aórtica crónica y que se observa en nuestro caso es la permeabilidad de la luz falsa, presente en un 36 a 41% de pacientes.^2^

Rylski et al. realizaron un estudio en 696 pacientes comparando los resultados de la cirugía de disección aórtica Stanford A crónica y aguda, y concluyeron que los pacientes con disección crónica tienen con mayor frecuencia el antecedente de una cirugía cardíaca previa (37% vs 9%) y que el 70% de ellos se mostraron asintomáticos al inicio de la disección. La denervación del sistema nervioso simpático cardíaco y la presencia de adherencias mediastínicas que “estabilizan” la disección explicarían estos hallazgos.^4^

Por último, la historia de revascularización coronaria es frecuente en casos de disección aórtica con cirugía cardíaca previa. Rylski et al, Stanger et al y Hirose et al. estudiaron la disección aórtica Stanford A después de una cirugía cardíaca, y encontraron que la cirugía de revascularización coronaria estuvo presente en el 68%, 53% y 58% de estos pacientes, respectivamente.^5^^,^^6^^,^^7^

## Conclusión

Un paciente con disección aórtica Stanford A crónica puede permanecer asintomático durante años. La reagudiza-ción del cuadro se asocia con un rápido incremento del diámetro de la aorta. Además, la disección aórtica Stanford A crónica está asociada a cirugía cardíaca previa, generalmente revascularización coronaria.

## Financiación:

Ninguna
